# Spontaneous Radial Nerve Palsy Subsequent to Non-Traumatic Neuroma

**DOI:** 10.5812/traumamon.9851

**Published:** 2013-08-13

**Authors:** Adel Ebrahimpour, Shahram Nazerani, Reza Tavakoli Darestani, Salim Khani

**Affiliations:** 1 Taleghani Hospital, Shahid Beheshti University of Medical Sciences, Tehran, IR Iran; 2 Tehran University of medical Sciences, Tehran, IR Iran; 3 Imam Hossein Hospital, Shahid Beheshti University of Medical Sciences, Tehran, IR Iran

**Keywords:** Radial Neuropathy, Neuroma, Wrist, Fingers

## Abstract

**Introduction:**

Spontaneous radial palsy is a not rare finding in hand clinics. The anatomy of the radial nerve renders it prone to pressure paralysis as often called “Saturday night palsy”. This problem is a transient nerve lesion and an acute one but the case presented here is very unusual in that it seems this entity can also occur as an acute on chronic situation with neuroma formation.

**Case Presentation:**

A 61 year-old man presented with the chief complaint of inability to extend the wrist and the fingers of the left hand which began suddenly the night before admission, following a three-week history of pain, numbness and tingling sensation of the affected extremity. He had no history of trauma to the extremity. Electromyography revealed a severe conductive defect of the left radial nerve with significant axonal loss at the upper arm. Surgical exploration identified a neuroma of the radial nerve measuring 1.5 cm in length as the cause of the paralysis. The neuroma was removed and an end-to-end nerve coaption was performed.

**Conclusions:**

Complete recovery of the hand and finger extension was achieved in nine months.

## 1. Introduction

The radial nerve is the most common nerve in the upper extremity to be affected by trauma (1). The most frequent etiologic agent is the fracture of the middle third of the humerus (2). Some other reasons for the palsy of this nerve include entanglement of the nerve within the fibrotic bands of the external head of triceps muscle, iatrogenic trauma during surgical procedures, ganglion in the elbow area and severe muscular effort, etc (3-6).The trauma might be in the form of pulling, compression and in rare cases, amputation (7). These traumas usually occur distal to the branches innervating the triceps muscle. Despite numerous reports about radial nerve palsy secondary to the various reasons mentioned above, there are only a limited number of reports in relation to spontaneous palsy of the radial nerve (8, 9). The present report describes a case of spontaneous radial nerve palsy subsequent to non-traumatic neuroma.

## 2. Case Presentation

A 61-year-old married man referred to the orthopedic clinic of our hospital, complaining of inability to extend his left hand fingers and wrist. The patient was admitted in the orthopedic ward. He reported a gradual onset of pain and tingling sensation in the affected extremity with three week duration; however, the night before admission while watching TV he had rested his hand under his head for a long period of time. In the morning, he found that he was unable to extend the wrist and fingers of his left hand. The patient had no history of chronic metabolic or inflammatory diseases, diabetes, hypertension or sensory-motor and balance disturbances; in addition, he did not have any history of trauma. He did not report a history of smoking or use of a specific medication. Clinical examinations revealed normal systemic functions. Examination of the affected extremity revealed complete motor palsy of the extensors of the wrist and fingers. The sensation of the dermatome of the distal radial nerve to the wrist was nonexistent. The pulse, temperature and color of the affected extremity were completely symmetrical and normal compared to the unaffected extremity. Laboratory examinations: Plain radiographs of the affected extremity did not reveal any signs of previous fractures, foreign bodies or masses with external pressure. Electromyography revealed poor and absent nerve impulse conduction of the radial nerve in relation to movement and sensation, respectively. In addition, evaluation of brachioradialis, extensor carpi radialis longus and extensor muscles of the fingers showed very poor or absent motor unit action potential (MUAP). In summary, electromyography demonstrated severe lesion of the radial nerve of the left extremity in the proximal area of the arm, along with significant axonal loss without any evidence of regeneration in the muscles mentioned.

**Figure 1.**
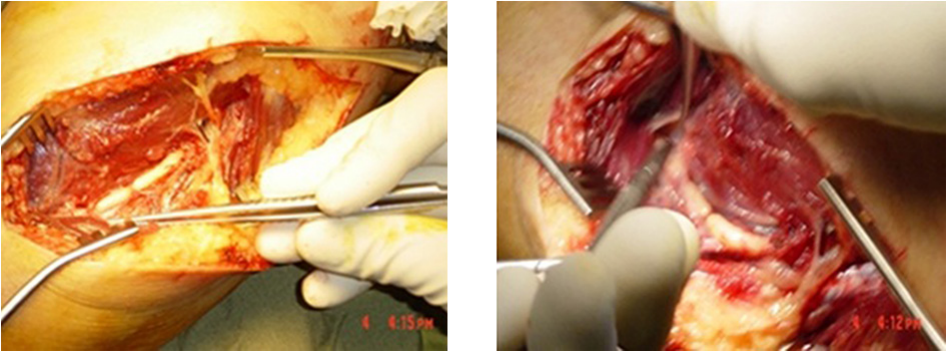
Macroscopic View of the Lesion

**Figure 2.**
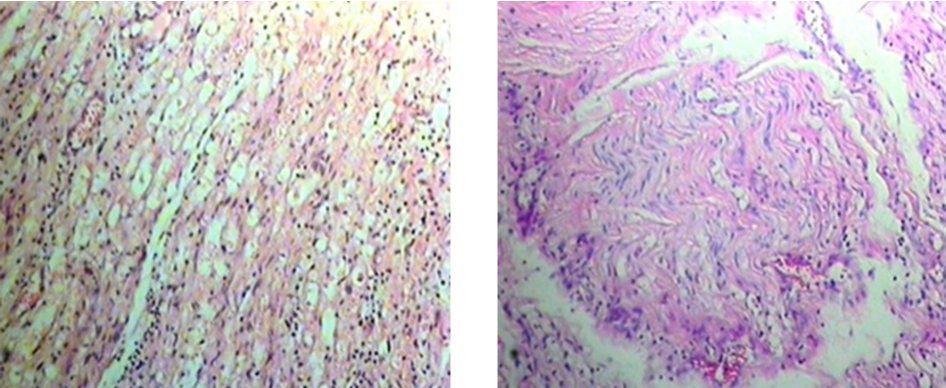
Microscopic View of the Lesion

Surgery was done under general anesthesia and tourniquet control and the radial nerve was explored. The surgical findings were two sausage-shaped areas at the distal third of the nerve (Figure 1). A neuroma (1.5 cm in length) was excised and the two ends of the nerve were coapted under magnification using 6-0 prolene suture. The limb was placed in a splint at 60 degrees flexion with the shoulder in adduction. The excised nerve segment was submitted for pathological evaluation, which yielded a diagnosis of neuroma (Figure 2). The patient achieved complete recovery of upper extremity movements after 9 months.

## 3. Conclusions

The radial nerve might be traumatized at different parts of its track; however, the most susceptible part is at the middle of the humerus (10). Since the majority of injuries are distal to the branches innervating the triceps muscle, generally the clinical manifestations of these injuries will appear in the form of wrist drop and inability to extend the fingers. Sleep palsy or Saturday-night palsy usually occurs after the nerve is subjected to pressure at its exit from the axillary region, brachio-axillary angle or where the nerve circles around the humerus, resulting in its sudden palsy (11). In this kind of palsy, usually there is no history of previous nerve problem and the electromyography results are normal. In the case presented here, sleep palsy is not considered as the first differential diagnosis regarding a 3-week history of tingling sensation in the affected upper extremity, despite the sudden onset of palsy and its occurrence after a long rest on the affected extremity. Radial nerve palsy after intense muscular effort may be considered for some patients. In one report, a 41 year-old Yemeni man had wrist drop and inability to open the fingers along with a sensory disturbance of the back of the hand after a day of heavy muscular effort (3). Electromyography of the patient showed normal impulse conduction and action potential at 0.1-ms excitation with 150-V electric potential. Patient electromyography findings showed disturbances in the nerve impulse conduction, therefore muscular effort was not an etiologic factor. In another case report, an 18 year-old girl presented with the same symptoms. Approximately 8 years previous to the incident, during an operation a pin had been placed for a supracondylar fracture and then severe fibrosis and pressure of the pin had resulted in palsy (12). In cases similar to the present case, surgical exploration of the nerve at a location where electromyography shows pathologic signs will solve the mystery. There are similar reports of neuroma of the radial nerve due to repeated trauma (13, 14). In a similar case in 2006, neuroma of the radial nerve was discovered with the ultrasound technique of the entire length of the nerve, proximal to the elbow fossa, which was confirmed by pathologic evaluation (15). Although the patient in the present report had no clear history of trauma or previous fracture, it seems that repeated micro trauma in the regions of the nerve which under pressure may have precipitated the disruption of nerve impulse conduction.
